# Renal tubular dysfunction measured by N-acetyl-beta glucosaminidase/Creatinine activity index in children receiving antiepileptic drugs: a randomized controlled trial

**DOI:** 10.1186/1824-7288-37-21

**Published:** 2011-05-14

**Authors:** Mojgan Mazaheri, Afshin Samaie, Vahid Semnani

**Affiliations:** 1 Pediatric nephrologist, Assistant professor, Semnan University of Medical Sciences, Semnan, Iran; 2 Neurologist, Assistant professor, Semnan University of Medical Sciences, Semnan, Iran; 3 Assistant professor, Semnan University of Medical Sciences, Semnan, Iran

## Abstract

To evaluate renal side-effects of anti-epileptic medication by valproate (VPA) and carbamazepine (CBZ), we performed a prospective study to assess renal tubular function by measuring *N*-acetyl-β glucosaminidase (NAG)/Cr activity index in epileptic children. The study was conducted on 112 children who were diagnosed with epilepsy (28 patients were observed before treatment with anti-epileptics, 28 children were administered VPA, 28 children were treated with CBZ, and 28 healthy children were selected age &sex matched for). An especial NAG assay kit was used for quantitative measuring of NAG in patient urine samples. The patients receiving VPA exhibited higher rate of NAG activity compared with the two groups which not receiving anti-epileptic drugs. Measurement of urinary NAG/Cr index in the children who received CBZ also, was significantly higher than those who were not administered anti-epileptic drugs. The measurement of NAG/Cr index in the VPA group was significantly higher than that in the CBZ group (NAG index: 2.75 versus 1.71). Children on anti-epileptic treatment with VPA or CBZ might demonstrate signs of renal tubular dysfunction, reflected by NAG/Cr activity index. This side effect can be potentially more occurred following VPA administration.

## Introduction

The use of common antiepileptic drugs can potentially result in some toxic reactions such as dermatitis, nephritis, hepatitis, as well as severe anemia. Some of these impairments are characterized by the changes of enzymes that one of the most applicable enzymes secreted in urine is *N*-acetyl-β glucosaminidase (NAG) that its activity is considered as a sensitive marker for diagnosis of renal tubular impairment in various disease states and its clinical diagnostic efficiency has been approved in some researches [1,2]. NAG is a hydrolytic enzyme with a molecular weight of 130,000 to 140,000 daltons. It is normally not filtered at the glomerulus. NAG is a widely distributed lysosomal enzyme, located predominantly in the renal proximal tubules [3]. It has previously been reported that NAG activity in rat urine increases after exposure to nephrotoxic agents in animal models [4]. Recently, increased excretion of urinary NAG in patients who were treated with valproate (VPA) and carbamazepine (CBZ) has been also suggested in some human trials [5-7]. However, some others revealed that the increased excretion of tubular enzymes and proteins in children with epilepsy is most probably not due to side-effect of the anti-epileptic drugs, but to a physiological alteration associated with the epilepsy itself [8]. Totally, it seems that the influence of epilepsy itself or its therapeutic antiepileptic regimen on stimulating activity of urinary indicator enzymes and thus renal damages via increasing of this enzyme is already unknown. The aim of this study was to assess the effect of some antiepileptic drugs (VPA and CBZ) on NAG activity measured by NAG/Cr activity index in epileptic children. In fact, our study endpoint was to measure urinary NAG/Cr index in the children who received CBZ or VPA. Then we compared increased excretion of tubular NAG enzyme in children treating these common anti-epileptic treatment regimens and compared it with those who were not administered anti-epileptic drugs.

## Methods

### Study population

The study was conducted on 112 children (63 boys and 49 girls, mean age 8.30 ± 2.50 years, range 3-16 years) who were recently referred to the neurology clinic of Fatemieh hospital in Semnan and diagnosed with epilepsy. Child with the signs of renal dysfunction and those with the previous history of diabetes mellitus or liver diseases were excluded. Study children were divided into four groups: 1) the patients with the final diagnosis of epilepsy, but did not receive antiepileptic treatments (n = 28); 2) those with confirmed epilepsy received VPA (n = 28); 3) the patients with epilepsy treated with CBZ (n = 28); and 4) the control group consisted of 28 healthy children. The course of VPA varied between 6 and 48 months with an average of 20.5 ± 11.2 months. The VPA doses given to the patients were a minimum of 15 mg/kg/day, maximum 25 mg/kg/day, with an average of 20 ± 3 mg/kg/day. Also, the course of CBZ varied between 6 and 48 months with an average of 21.7 ± 12.6 months. The CBZ doses given to the patients were a minimum of 10 mg/kg/day, maximum 30 mg/kg/day, with an average of 24 ± 4 mg/kg/day. The mean duration of the two antiepileptic treatments was not different. None of the epileptic and healthy children were taking medication at the time of urine sampling. Study protocol was performed according to the principles of the Declaration of Helsinki and approved by the ethics committee of the Semnan University of Medical Sciences. Written informed consent was obtained from all parents.

### NAG evaluation

A 10 mL aliquot of urine collected after the first morning void was used for assay of urine creatinine and the enzyme. NAG activity was assessed by NAG/Cr ratio measuring. An especial NAG assay kit (using the Boehringer Mannheim kits, Germany) was used for the quantitative in vitro determination of NAG in patient urine samples. In this assay, it was used a specific substrate, 2-methoxy-4-(2'nitrovinyl)-phenyl 2-acetamido-2-deoxy-β-D-glucopyranoside (MNP-GlcNAc) that is hydrolyzed by NAG to produce 2-methoxy-4-(2'-nitrovinyl)-phenol product. The product formation is detected at 505 nm upon addition of an alkaline buffer. Blood creatinine levels were determined using an Olympus 5241 apparatus (Hamburg, Germany).

### Statistical analysis

Results were reported as mean ± standard deviation (SD) for the quantitative variables and percentages for the categorical variables. The groups were compared using the Mann Whitney U test for the continuous variables and the chi-square test (or Fisher's exact test if required) for the categorical variables. P values of 0.05 or less were considered statistically significant. All the statistical analyses were performed using SPSS version 16.0 (SPSS Inc., Chicago, IL, USA) for Windows.

## Results

**Figure 1:**
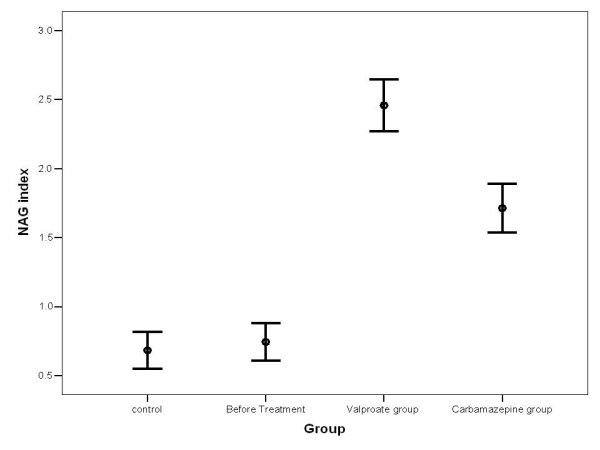
NAG index measurement in four study groups (presented as mean ± SD)

As shown in Table 1, there were no differences in male to female ratio as well as mean age across the four study groups. The NAG activity, assessed by the NAG/Cr index measurement in the groups of healthy children, patients group before receiving anti-epileptic therapy, and patients who received CBZ or VPA is presented in Figure 1. There is no significant difference in NAG activity between the control group and another non-treatment group (NAG index: 0.68 versus 0.73, p = 0.611). However, the patients who were receiving VPA exhibited higher rate of NAG activity compared with the two groups which not receiving anti-epileptic drugs (p < 0.001). Also, measurement of urinary NAG/Cr index in the children who received CBZ was significantly higher than those who were not administered anti-epileptic drugs (p < 0.001). Mean plasma concentrations of VPA and CBZ in these two treated patient groups were 68.7 ± 17.4 μg/mL and 5.4 ± 1.2 μg/mL, respectively. There was a significant correlation between the serum concentration of VPA and urinary NAG levels (r = 0.39, P < 0.01). There was also a significant correlation between the serum concentration of CBZ and NAG excretion (r = 0.46, P < 0.01). Regarding relationship between the duration of treatment and NAG index, this correlation was significant in the patients who received VPA (r = 0.56, P < 0.01) as well as in those who received CBZ treatment (r = 0.36, P < 0.01).

**Table 1:** Baseline characteristics of four study groups

*Study group*	*Number*	*Male/female ratio*	*Age*	
			Mean	SD
Control group	28	16/12	8.29	3.68
Before treatment	28	15/13	8.36	3.91
Valproate group	28	16/12	8.43	3.61
Carbamazepine group	28	16/12	8.14	3.34
P-value		0.998	0.979	

With regard to the difference in NAG/Cr index between the two treated groups, measurement of this index in the VPA group was significantly higher than that in the CBZ group (NAG index: 2.75 versus 1.71, p < 0.001). In each study group, the mean NAG/Cr index was statistically similar between the two genders (Table 2).

**Table 2:** Comparison of the NAG index measurement between the two genders in each group

*Study group*	*NAG index* *(total)*	*NAG index* *(Boys)*	*NAG index* *(girls)*	*P-value*
Control group	0.68 ± 0.34 ^*†^	0.73 ± 0.39^*†^	0.61 ± 0.25^*†^	0.332
Before treatment	0.74 ± 0.35	0.66 ± 0.36	0.80 ± 0.34	0.300
Valproate group	2.75 ± 0.48*	2.43 ± 0.52*	2.48 ± 0.43*	0.783
Carbamazepine group	1.71 ± 0.46^†^	1.69 ± 0.39^†^	1.74 ± 0.55^†^	0.791

Data are presented as mean ± SD

* and ^† ^indicate significant intergroup differences (p < 0.001)

## Discussion

Current study could confirm previous reports about an increased excretion of tubular NAG enzyme in children treating common anti-epileptic treatment regimens. Our study showed that mean value of NAG/Cr index was increased 3.7-fold following administration of VPA over the median values of the epileptic children before treatment. Elevation of this index after the treatment with CBZ was 2.3-fold following administration of VPA over the median values of epileptic ones before treatment. However, the measure of NAG/Cr index was not significantly different between the two untreated groups. The adverse impact of mono and combined therapies on NAG/Cr index and therefore on renal tubular function has been observed in several previous studies, while some others demonstrated the clinical efficiencies of these drugs in epileptic children. In a study by Otsuka et al. the level of urinary excretion of NAG was high in 29% of all patients, in 47% of VPA group, and in 38% of CBZ group. In their study, a significant positive correlation was observed between NAG/Cr index and serum concentration of VPA [9]. In another study by Tseng et al. VPA administration was accompanied with the highest incidence of abnormal urinary NAG/Cr index (in about 78% of treated children) that was significantly higher than that in the CBZ group with the incidence of 26%. However, the incidence of high urinary NAG index in the poly-therapy group and that in mono-therapy group was significantly similar [10]. These adverse effects were also approved in studies on animal models. Some experimental studies revealed high NAG level in rats with partial ureteral obstruction and hydronephrotic atrophy [11,12]. Contrarily, in Hurkacz et al. study, increasing of NAG/Cr activity index was shown with concomitant therapy, but that was in the normal range and therefore tubular dysfunction was not approved following their treatment schedule [7]. Also, in another study by Korinthenberg et al. those on anti-epileptic treatment with therapeutic drug levels demonstrated minor signs of tubular dysfunction [13]. In earlier investigations, it has been hypothesized that following administration and absorption of some anti-epileptic drugs, the lysosomal contents of the tubular cells might be secreted into the lumen of the urinary tract to eliminate indigestible residues that are left after hydrolysis of high molecular weight material recaptured from the glomerular filtrate [14]. In addition, NAG is a lysosomal hydrolasis that plays an important role in the catabolism of both glycoproteins and glycosaminoglycans [15-17]. This mechanism is mainly occurred in the proximal tubulus and therefore can be interpreted as an indicator of functional disturbance of tubuls [13]. Thus, according to our study results as well as other previous findings, NAG/Cr index can be used as a sensitive indicator of renal tubular disease activity, and it might be a suitable screening test for early diagnosis of renal disturbance.

In our survey, duration of treatment in all study groups was similar. It was suggested that distribution of NAG index of patients depended significantly on the length of therapy so that the level of urinary excretion of NAG was significantly higher in those who were taking long-term treatment (>10 years) with VPA, CBZ and combined therapy than those taking therapy shorter than 10 years [18]. Even, after 1 to 2 years of the beginning of treatment, these patients may show persistence of the changes found after 6 months of therapy [19]. Therefore, in the necessity of long-term administrating these drugs particularly VPA in epileptic children, a suitable dose of drug as well as appropriate treatment time should be primarily considered for minimizing their probable side effects.

## Conclusion

Based on the findings of this study, children on anti-epileptic treatment with VPA or CBZ might demonstrate signs of renal tubular dysfunction, reflected by NAG/Cr activity index. This side effect can be potentially more occurred following VPA administration.

## Competing interests

The authors declare that they have no competing interests.

## Authors' contributions

MM designed the study, selected the study groups, performed statistical analysis, & drafted the manuscript. AF referred the patients &carried out their treatment, VS carried out the laboratories assays. All authors have read and approved the final manuscript.
